# Three-Dimensional-Bioprinted Non-Small Cell Lung Cancer Models in a Mouse Phantom for Radiotherapy Research

**DOI:** 10.3390/ijms251910268

**Published:** 2024-09-24

**Authors:** Yikun Mei, Elena Lakotsenina, Marie Wegner, Timon Hehne, Dieter Krause, Dani Hakimeh, Dongwei Wu, Elisabeth Schültke, Franziska Hausmann, Jens Kurreck, Beatrice Tolksdorf

**Affiliations:** 1 Department of Applied Biochemistry, Institute of Biotechnology, Technische Universität Berlin, 10623 Berlin, Germany; yikun.mei@campus.tu-berlin.de (Y.M.); dani.hakimeh@charite.de (D.H.);; 2 Department of Radiation Oncology, Charité University Medicine Berlin, Corporate Member of Freie Universität Berlin and Humboldt-Universität Zu Berlin, 13353 Berlin, Germany; elena.lakotsenina@charite.de (E.L.); timon.hehne@charite.de (T.H.); franziska.hausmann@charite.de (F.H.); 3 Department of Product Development and Mechanical Engineering Design, Hamburg University of Technology, 21073 Hamburg, Germany; marie.wegner@tuhh.de (M.W.); krause@tuhh.de (D.K.); 4 Department of Pediatric Oncology and Hematology, Charité University Medicine Berlin, 13353 Berlin, Germany; 5 Department of Radiooncology, Rostock University Medical Center, 18059 Rostock, Germany; elisabeth.schueltke@med.uni-rostock.de

**Keywords:** 3D bioprinting, lung cancer, radiation therapy, mouse phantom, model system

## Abstract

Lung cancer continues to have one of the highest morbidity and mortality rates of any cancer. Although radiochemotherapy, in combination with immunotherapy, has significantly improved overall survival, new treatment options are urgently needed. However, preclinical radiotherapy testing is often performed in animal models, which has several drawbacks, including species-specific differences and ethical concerns. To replace animal models, this study used a micro-extrusion bioprinting approach to generate a three-dimensional (3D) human lung cancer model consisting of lung tumor cells embedded in human primary lung fibroblasts for radiotherapy research. The models were placed in a mouse phantom, i.e., a 3D-printed mouse model made of materials that mimic the X-ray radiation attenuation rates found in mice. In radiotherapy experiments, the model demonstrated a selective cytotoxic effect of X-rays on tumor cells, consistent with findings in 2D cells. Furthermore, the analysis of metabolic activity, cell death, apoptosis, and DNA damage-induced γH2AX foci formation revealed different results in the 3D model inside the phantom compared to those observed in irradiated models without phantom and 2D cells. The proposed setup of the bioprinted 3D lung model inside the mouse phantom provides a physiologically relevant model system to study radiation effects.

## 1. Introduction

Despite a sustained decline in incidence and mortality rates over the past decade, lung cancer remains the leading cause of cancer-related mortality [1]. In terms of incidence, lung cancer is the second most common cancer in males after prostate cancer and in females after breast cancer. The strikingly high mortality rate, which accounts for over 20% of all cancer deaths each year, exceeds the mortality rates for prostate, breast, and colorectal cancers combined [2]. The majority of cases are due to non-small cell lung cancer (NSCLC). Radiotherapy (RT), often in combination with chemotherapy and adjuvant immunotherapy, plays a pivotal role in the treatment of this disease, with more than half of patients receiving treatment for both curative and palliative purposes [3].

Evidence-based medicine indicates that three-quarters of lung cancer patients require radiotherapy [4]. The development of new radiotherapy techniques has made it possible to deliver targeted radiation to tumors with greater precision while reducing radiation exposure to surrounding normal tissues. These technological advances have expanded the indications for radiotherapy in lung cancer, resulting in improved survival and reduced toxicity [5]. Moreover, despite the use of the same technology, distinct radiotherapy protocols must be tailored for patients at different stages of NSCLC and for individuals with different lesion characteristics. Physiologically relevant models of lung cancer can support the development of improved radiotherapy treatments.

The biological processes that follow radiotherapy for lung cancer include DNA damage, inflammation, and fibrosis [6]. The understanding of these processes is based on clinical observations and experimental studies in in vitro, ex vivo, and animal models. These studies provide critical insights into pulmonary radiation responses. In addition, the development of advanced small animal irradiation devices is improving the precision and accuracy of dose delivery in mice, thereby increasing their utility in preclinical studies of lung cancer radiotherapy treatments [7].

Nevertheless, accurately modeling radiation responses in mice remains a significant challenge, and current murine radiation models have inherent limitations. For example, respiratory motion complicates the precise targeting of thoracic sites during treatment. Differences between mouse strains introduce bias when extrapolating findings to humans. Additionally, the total lung capacity of a mouse is only about 1 mL, compared to approximately 6 L in humans, which is significantly larger. This discrepancy presents inherent challenges when delivering small beam sizes, typically in the millimeter range. Clearly, certain aspects of human radiation responses cannot be fully replicated in mice, not to mention the ethical controversies surrounding animal experimentation [7,8]. Moreover, the use of mouse tumor models is further complicated by the lengthy protocols, high costs, and operational complexities inherent in such models [9].

In recent years, the development of 3D bioprinting technology has emerged as a promising strategy for developing human-based models to study human pathophysiology [10]. This advanced technology facilitates the precise arrangement of diverse human cells within three-dimensional structures, with the objective of reproducing the intricacies of tissues in vitro. The creation of a structurally defined cell-laden matrix allows the combined benefits of multiple human cells distributed or arranged in a pre-designed 3D spatial environment [11,12]. It is noteworthy that bioprinting has demonstrated considerable potential in the dynamic field of cancer biology. The inherent flexibility of this technology allows precise tuning of the stiffness of the extracellular environment, integration of multiple cell types, and design of complex three-dimensional structures. The majority of bioprinted cancer models are derived from human cancer cells. Even when non-cancer cells are integrated into the model, they are typically of human origin, overcoming some of the limitations of traditional animal models [13].

Based on the above, our study employed an extrusion-based printing method to bioprint a fusion of normal human primary lung fibroblasts and NSCLC cells. To simulate the process of X-ray penetration through mouse skin, bone, and organs to reach the tumor site, materials approximating the X-ray radiation attenuation rates found in mice were used in a mouse phantom [14]. The phantom was used to support the 3D-printed tumor model. Radiation therapy results showed a clear selective cytotoxic effect of X-rays on the 3D lung cancer model, which was consistent with the 2D cell results. Furthermore, by co-culturing normal cells in the outer layers of the 3D model and placing the 3D model in the mouse phantom, which better simulates the actual treatment process, the analysis of metabolic activity, cell death, apoptosis, and DNA damage-induced γH2AX foci formation showed different results in the 3D model compared to 2D cells. In addition, future studies involving more complex radiation effects such as cellular senescence, hypoxia, and necrosis will benefit from the long-term culture capability of the 3D model.

## 2. Results

### 2.1. Development of a 3D-Printed Human Lung Cancer Model for Radiotherapy Experiments

Previously, we successfully developed a basic 3D-printed human lung cancer model for radiotherapy studies involving broad-beam and micro-beam techniques [15]. Based on these findings, the 3D lung model and experimental setup were further improved in this study to better reflect physiological conditions.

**Figure 1:**
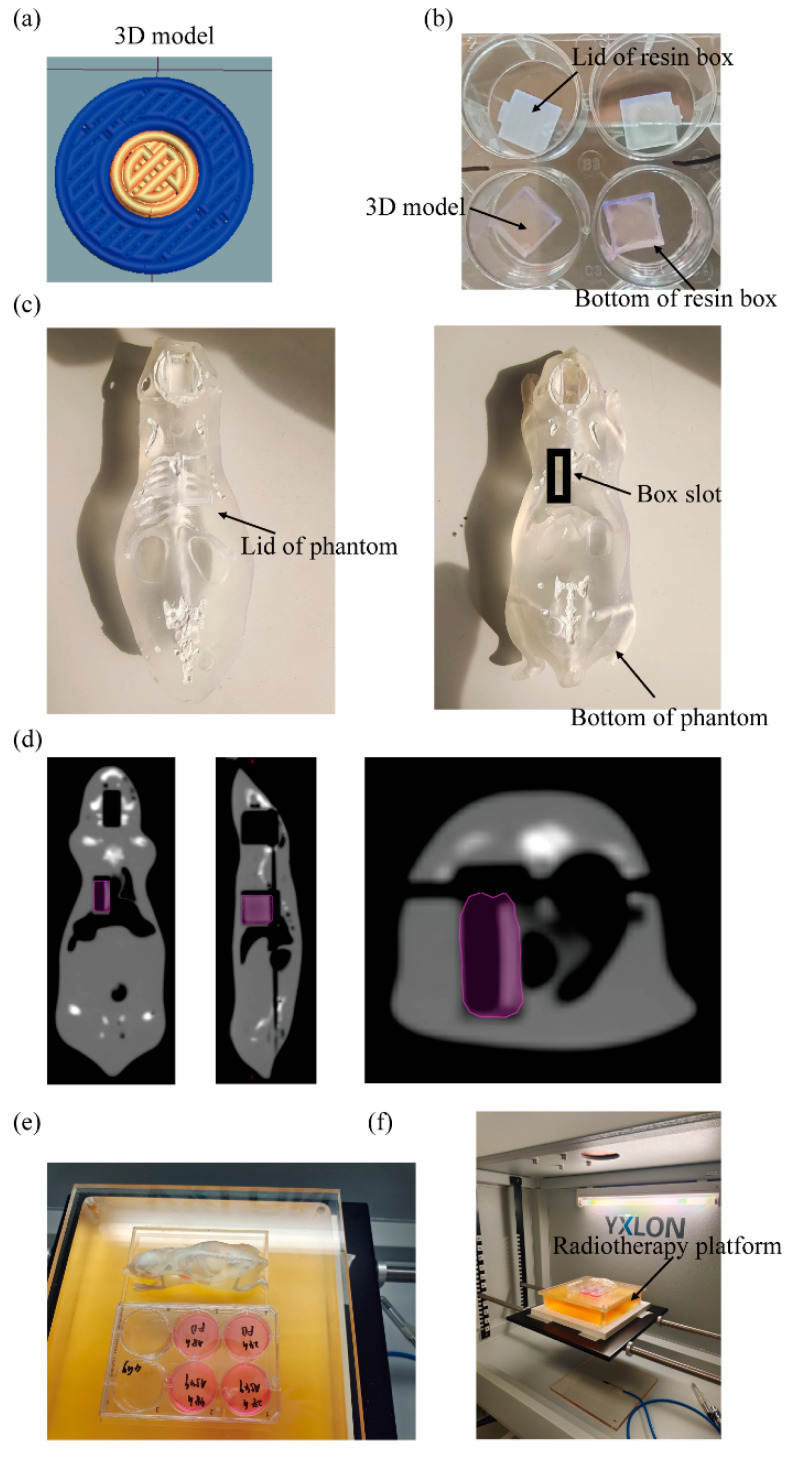
Design of the 3D-printed human lung cancer model and arrangement of the radiotherapy equipment. (**a**) Schematic representation of the 3D model. (**b**) Three-dimensional models in resin boxes. (**c**) Upper and lower parts of the mouse phantom. (**d**) Coronal, sagittal, and axial CT images of the mouse phantom. The purple area highlights the thoracic slot where the resin box containing the 3D model can be placed in the mouse lung. (**e**) Experimental setup of 2D cell culture plates and the mouse phantom for radiation. (**f**) The entire internal irradiation area of the radiotherapy device.

The improved model was designed to consist of a central cancerous part containing A549 cells surrounded by normal tissue containing normal human primary lung fibroblasts (Figure 1a).

The model has a height of 0.6 mm, a diameter of 9 mm, and a central circular disc diameter of 4.5 mm. For micro-extrusion bioprinting of the models, the cells were embedded in a hydrogel consisting of alginate and gelatin. After printing, the models were cultured for one week to allow them to recover from the printing process. On day eight, the models were placed in a biocompatible resin box, inner dimensions 11 mm × 11 mm × 2.4 mm (Figure 1b), which was then inserted into the thoracic slot of a previously published mouse phantom [14] for irradiation (Figure 1c). In the CT images of the mouse phantom, the internal structure can be observed (Figure 1d), with the purple area indicating the position of the lung cancer model, which is located precisely where the lung would be in a real mouse. The upper part of the lung corresponds to the sternum area, where the white signal represents the calcium component, simulating bone. The lower part of the lung corresponds to the abdominal cavity, which is free of material. Together, these structures simulate the process of radiation passing through the skin, bone, and normal lung tissue to irradiate the lungs. The irradiation platform has dimensions of 17 cm × 13 cm, which is sufficient to accommodate two well plates or one well plate and the mouse phantom (Figure 1e). The entire experimental process is illustrated in the graphical abstract.

### 2.2. Comparison of Dose Distributions of the Mouse Phantom with Those of an In Vivo Mouse Model

**Figure 2:**
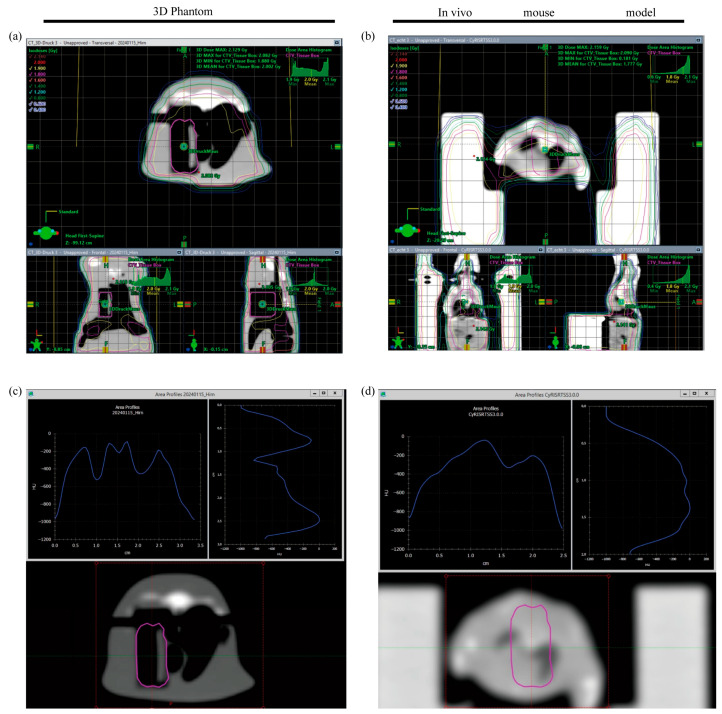
Dose distribution (marked by colored isodose curves) as well as mean irradiation doses were comparable between the 3D phantom (**a**) and the in vivo mouse model (**b**). Investigation of density histograms showed differences according to anatomical localization (e.g., lower Houndsfield units in lung tissue) (**c**,**d**). Clinical target volume (pink contour) was delineated by hand on a CT scan of the 3D phantom and manually transferred to a CT scan of the in vivo mouse model [16].

For analysis of dose distributions, the tissue box was manually contoured as a clinical target volume (CTV) on a simulated CT scan of the 3D mouse phantom and compared to pre-existing data of an in vivo mouse model. The planned dose to the CTV was 2 Gy with an ICRU conform dose distribution (95–107% of the prescribed dose in target volume). The dose maximum for the CTVs were 2.082 and 2.090 Gy for the 3D phantom and the in vivo mouse model, respectively (Figure 2a,b). While Monitor units were kept similar in both irradiation plans at 217.1, the minimal dose was 1.88 Gy (3D phantom) and 0.181 Gy (in vivo mouse model). Similarly, the mean dose was slightly lower in the in vivo mouse model compared to the 3D phantom (1.77 Gy vs. 2.002 Gy).

For the 3D phantom, the density histogram shows decreased density over the lungs, comparable to the in vivo mouse model (Figure 2c,d). These results indicate that compared to a standard in vitro irradiation, irradiation of 3D models in the phantom can better mimic differences in tissue density and thus potentially better reflect the clinical dose distribution.

### 2.3. Cell Viability and Metabolic Activity of 2D Cells and 3D Models after Radiotherapy

An initial assessment was performed to evaluate the response of 2D cells to irradiation seeded in well plates. To account for potential temporal variations in radiation effects after irradiation, assessments were performed 24 and 48 h after irradiation. In accordance with the cellular composition of the 3D model, A549 cells, representing NSCLC cells, and normal human primary lung fibroblasts (NHLFb), representing normal cells, were irradiated at doses of 4 Gy and 8 Gy, respectively, as the commonly used dose for a single fraction of radiotherapy in clinical practice typically ranges from 2 to 10 Gy.

**Figure 3:**
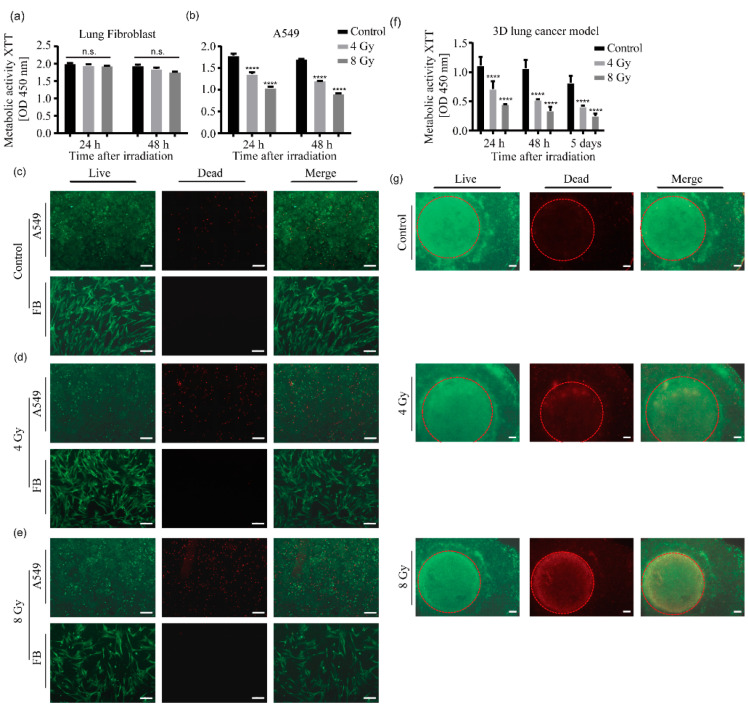
Cell viability and metabolic activity of 2D cells and 3D models after irradiation. The metabolic activity of 2D lung fibroblasts (FB) (**a**) and A549 cells (**b**) was measured by a tetrazolium hydroxide salt (XTT) assay at the indicated time points after irradiation with 4 or 8 Gy, respectively. The control groups were kept under the same conditions as the two experimental groups, except that they were not irradiated. Data from three independent experiments are presented as mean ± standard deviation, **** *p* < 0.0001. (**c**–**e**) The viability of 2D cells was evaluated for the non-irradiated control (**c**), 4 Gy irradiation group (**d**), and 8 Gy irradiation group (**e**), at 24 h post-radiotherapy using calcein-AM and ethidium homodimer-1 staining. Under fluorescence microscopy, viable cells appeared green, while dead cells appeared red. Scale bar: 200 µm. (**f**) The metabolic activity of the 3D lung cancer models was measured using an XTT assay at 24 h, 48 h, and 5 days after irradiation, and the data were plotted for radiation doses of 4 Gy and 8 Gy. The control group was maintained under the same conditions as the two experimental groups except that it was not irradiated. Data from three independent experiments are presented as mean ± standard deviation, **** *p* < 0.0001. (**g**) Cell viability staining of the 3D lung cancer models was conducted at 24 h post-radiotherapy using calcein-AM and ethidium homodimer-1 staining. The cancer part containing A549 cells is marked with a red circle. Scale bar: 500 µm.

The viability of lung fibroblast cells did not differ significantly from that of the control group at 24 and 48 h after irradiation (Figure 3a) and remained at a consistently high level. In contrast, the viability of A549 cells decreased significantly after radiotherapy, with a more pronounced effect observed at the 8 Gy dose compared to the 4 Gy dose (Figure 3b). In addition, a slight difference in effect was observed between the 24-h and 48-h post-irradiation assessments.

Figure 3c illustrates the rapid proliferation of A549 cells covering the entire field of view. A few dead cells are observed in the control group. In contrast, lung fibroblast cells exhibited a relatively slow proliferation rate, with minimal gaps between cells and almost no evidence of cell death in the field of view. In Figure 3d,e, lung fibroblast cells showed minimal evidence of cell death after irradiation. In stark contrast, a significant number of dead cell signals were observed in the irradiated A549 cells. Quantification of the dead cells by ImageJ (version 1.54j) revealed a higher proportion of dead cells in the 8 Gy group, with a slight increase in the number of dead cells at 48 h compared to 24 h (Figure S1). These findings are consistent with the results of the cell viability assays (Figure 3a,b).

In comparison to the direct irradiation of two-dimensional cells in well plates, the 3D models irradiated inside of the mouse phantom were evaluated. Given that the therapeutic effect on cells in this radiation mode may be less pronounced than that observed with direct irradiation of cells in well plates, measurements were conducted at multiple time points. As illustrated in Figure 3f, the overall cell viability exhibited a gradual decline at 24 h, 48 h, and 5 days post-irradiation. This decrease was observed to correlate with the administered radiation doses, demonstrating a dose–response relationship. The group that was exposed to 8 Gy exhibited the most pronounced decline in cell viability, reaching a level less than 50% of that observed in the control group. This result reflects the collective cell viability of the two cell types within the model.

To evaluate the two cell types in different regions of the model (the central tumor area and the surrounding normal lung fibroblasts), cell cytotoxicity staining was performed. As illustrated in Figure 3g, the number of dead cell signals observed in both the central and peripheral areas of the control group was minimal. In contrast, the central field of view of both irradiated groups exhibited bright areas of dead cells, with a pronounced difference between the central and peripheral areas observed in the 8 Gy dose group. These results demonstrate that tumor cell viability and metabolic activity are reduced shortly after irradiation, while normal lung fibroblasts are minimally affected.

### 2.4. Radiotherapy Induces DNA Double-Strand Breaks in 2D Cells and 3D Models

To identify DNA double-strand breaks, the DNA damage marker γH2AX was used. At the chromatin level, the localization and amplification of the damage signal are characterized by the phosphorylation of histone H2AX. This process is enhanced by the three main kinases involved in DNA double-strand breaks, which can be labeled with phospho-specific antibodies, forming nuclear substructures known as γH2AX foci [17].

**Figure 4:**
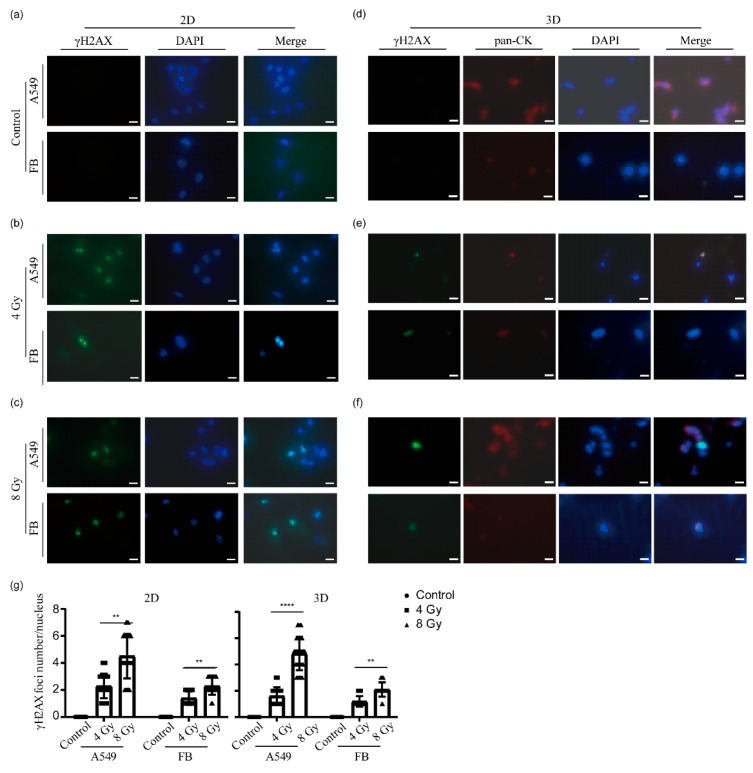
γH2AX immunofluorescence staining of irradiated 2D cells and 3D models. (**a**–**c**) Immunostaining images of A549 cells and lung fibroblasts (FB) from the unirradiated control (**a**), 4 Gy irradiation group (**b**), and 8 Gy irradiation group (**c**). Cells were fixed 24 h after irradiation. γH2AX staining (green channel) indicates DNA double-strand breaks. DAPI was used for nuclear counterstaining (blue channel). Scale bar: 20 µm. (**d**–**f**) Immunohistochemical staining images of 3D lung cancer models from the non-irradiated control group (**d**), 4 Gy irradiation group (**e**), and 8 Gy irradiation group (**f**). The models were fixed, dehydrated, and paraffin-embedded 24 h after irradiation, followed by sectioning at 16 µm thickness. The sections were then subjected to immunostaining. γH2AX staining (green channel) indicates DNA double-strand breaks. The samples were also stained with antibodies against pan-cytokeratin (pan-CK) to confirm their identity as epithelial cells (red channel). DAPI was used for nuclear counterstaining (blue channel). Scale bar: 20 µm. (**g**) After treatment with different radiation doses, the average number of γH2AX foci was determined in the nuclei of randomly selected A549 cells and fibroblasts in 2D, as well as in regions of A549 cells and fibroblasts within 3D model sections. Data from three independent experiments are presented as mean ± standard deviation, ** *p* < 0.01, **** *p* < 0.0001.

First, A549 cells and normal lung fibroblasts were seeded in 48-well plates and irradiated at doses of 4 Gy and 8 Gy, respectively. A non-irradiated group served as a negative control. The cells were fixed and stained 24 h after irradiation. As illustrated in Figure 4a, as expected, no γH2AX foci signals were observed in either cell type in the control group. In contrast, the 4 Gy groups exhibited approximately two γH2AX foci per nucleus (Figure 4b). In the 8 Gy group, a greater number of γH2AX foci were observed in the nuclei of both cell types, with the entire nuclear area displaying a dispersed distribution of γH2AX foci. Although the number of γH2AX foci varied between cells, statistical analysis of multiple randomly selected cells indicated that the number of γH2AX foci in the 8 Gy group was approximately twice that in the 4 Gy group, with statistically significant differences (Figure 4g).

For the 3D models, 24 h post-radiotherapy, the models were fixed, dehydrated, paraffin-embedded, and sectioned into 16 µm paraffin sections. Subsequently, the sections were subjected to immunohistochemical analysis for γH2AX. To distinguish between the two cell types in different regions (A549 tumor cells in the central part and fibroblasts in the peripheral part), red fluorescence representing pan-cytokeratin (pan-CK) was employed to label A549 cells. As illustrated in Figure 4d, as expected, neither the peripheral lung fibroblasts nor the central A549 cells in the control group exhibited γH2AX signals. Following exposure to a 4 Gy dose of radiation, A549 cells exhibited γH2AX foci. However, in fibroblasts, although the γH2AX signal completely overlapped with DAPI, multiple γH2AX foci did not form (Figure 4e). Following irradiation with 8 Gy, multiple γH2AX foci were observed in the nuclei of A549 cells, a result consistent with those obtained from 2D cell cultures. However, in fibroblasts, the number of γH2AX foci was relatively low, and the proportion of cells positive for γH2AX was minimal. In conclusion, the expression of γH2AX in the tumor portion of the 3D model was found to be largely consistent with that observed in 2D A549 cells.

### 2.5. Radiotherapy Induced Apoptosis and LDH Release in 2D Cells and 3D Models

Ionizing radiation induces DNA damage in cancer cells, which, if unrepaired, leads to cell death via mitotic cell death often resulting in apoptosis and cell necrosis. Therefore, we investigated the apoptotic marker cleaved caspase-3. Immunostaining results in 2D cell cultures demonstrated that lung fibroblasts exhibited minimal cleaved caspase-3 signals at 24 h after 4 Gy and 8 Gy irradiation (Figure S2b,c). A549 cells also exhibited minimal levels of cleaved caspase-3 signal at 4 Gy and 8 Gy irradiation doses (Figure S2b,c).

In contrast, the effect of radiotherapy on apoptosis in 3D cell models was investigated using histological section staining. Cleaved caspase-3 staining revealed that some pan-CK-labeled A549 cells exhibited apoptotic signals within the field of view at the 4 Gy dose (Figure S2e), whereas an increase in apoptotic signals was observed at the 8 Gy dose (Figure S2f). Lung fibroblasts did not show any apoptotic signals under non-irradiated and 4 Gy irradiation conditions. However, a minimal amount of cleaved caspase-3 signal was observed at the 8 Gy dose. In general, the degree of X-ray-induced apoptosis in the 3D lung cancer model was significantly less than that observed in 2D cells, with lung fibroblasts exhibiting the most notable difference. To determine whether the induction of necrosis may also contribute to the decreased viability after irradiation we also measured lactate dehydrogenase (LDH) release. Irradiation at 8 Gy significantly increased LDH release from 2D A549 cells and the 3D model compared to the control and 4 Gy groups (Figure S3). The release of LDH from 2D lung fibroblasts in response to radiotherapy was not observed. This suggests that radiotherapy affects tumor cells and normal cells differently.

### 2.6. Influence of the Mouse Phantom on Irradiation Effects

**Figure 5:**
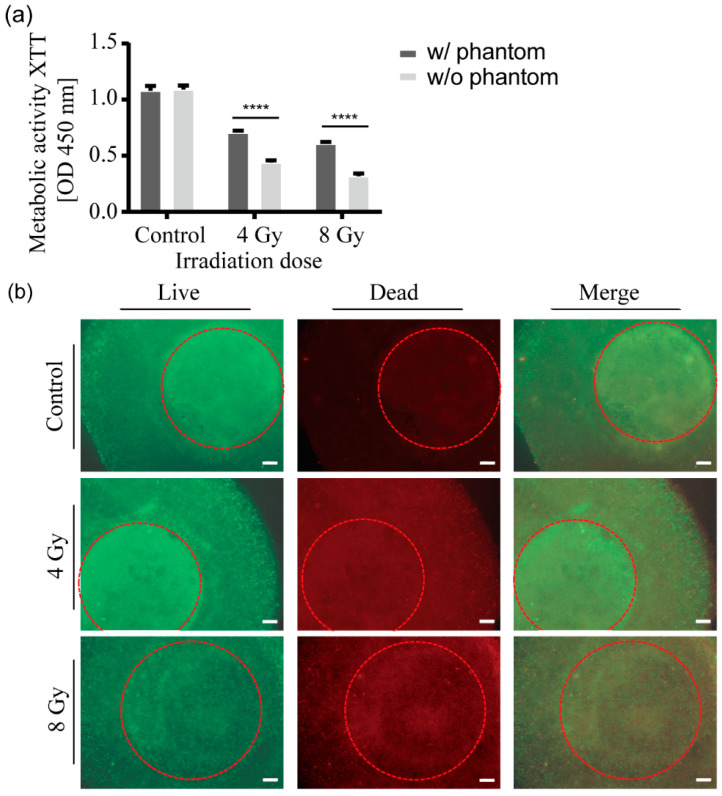
Cell viability and metabolic activity of 3D models after irradiation without the mouse phantom. (**a**) The metabolic activity of the 3D lung cancer models placed in a 12-well plate was measured using an XTT assay 24 h after irradiation with 4 or 8 Gy, respectively. The control group was maintained under the same conditions as the two experimental groups except that it was not irradiated. Data from three independent experiments are expressed as the mean ± standard deviation, **** *p* < 0.0001. (**b**) Cell viability staining of the 3D lung cancer models placed in a 12-well plate 24 h after irradiation using calcein-AM and ethidium homodimer-1 staining. Under fluorescence microscopy, viable cells appeared green, while dead cells appeared red. The cancer part containing A549 cells is marked with a red circle. Scale bar: 500 µm.

The mouse phantom was selected as a model to simulate the process of radiation passing through the skin, bones, and other organs of an animal to reach the tumor site. The CT scan above demonstrates that the intensity of radiation is attenuated as it passes through the phantom (Figure 2). To investigate whether the placement of the 3D lung models in the phantom had any effect on cell survival, 3D models irradiated inside the phantom were compared with 3D models placed directly in a 12-well plate. The total dose of irradiation was identical in both cases. As illustrated in Figure 5a, the cell viability in the phantom-free group was significantly lower than that of the 3D models in the mouse phantom at both 4 Gy and 8 Gy irradiation doses. Even at the 4 Gy dose, cell viability was reduced to less than half of the control.

Cell cytotoxicity staining was performed to evaluate the two cell types in different regions of the model (central tumor area and surrounding normal lung fibroblasts). Figure 5b shows 3D models 24 h after direct irradiation in a 12-well plate. As expected, the number of dead cell signals observed in both the central area and the peripheral areas of the control group was minimal. However, in stark contrast to the observations of the models in the phantom (Figure 3g), in both the 4 Gy and 8 Gy dose groups, the dead cells in the tumor region and the surrounding healthy region are indistinguishable, and there is no clear boundary between the two regions. The difference in staining results compared to Figure 3g is very noticeable. These results suggest that by placing the 3D model inside the mouse phantom, more physiological conditions can be created.

## 3. Discussion

The tumor microenvironment (TME) is defined as the complex interplay of tumor cells, healthy cells, immune cells, cytokines, and other components. The interactions between these components can be classified as either anti-tumor or pro-tumor, which ultimately determine the trajectory of treatments [18]. Consequently, interest has gradually shifted from single tumor cell models [19] to more complex models. In the context of lung cancer models used for radiotherapy, the relationship between normal tissue cells and tumor cells becomes particularly critical, as radiation not only targets tumor cells but also exerts cytotoxic effects on normal tissue cells.

In order to print two distinct cell types in disparate regions of the model, a previously validated hydrogel formulation was selected [15]. The combination of gelatin and alginate for extrusion-based bioprinting has become a well-established method for the construction of three-dimensional organ models. Gelatin is a widely used material due to its high biocompatibility, non-immunogenicity, and cell-friendly binding domains [20]. While alginate is minimally cytotoxic, it lacks cell adhesion properties. However, it can be crosslinked with calcium salts after printing [21]. For example, in 2021, Berg et al. developed a 3D lung model using a hybrid gelatin/alginate hydrogel to study influenza virus A infections [22]. Similarly, Kang et al. employed a gelatin/alginate hydrogel bioink to generate multilayered composite scaffolds that mimic the follicular microenvironment, including the stratum corneum and dermis [23]. With a similar hydrogel composition, Hiller et al. printed human HepaRG liver cells to evaluate their suitability for studies on adeno-associated virus (AAV) vector transduction and human adenovirus 5 (hAdV5) infection [24].

To reduce reliance on live animals while still recapitulating physiological conditions, the use of mouse phantoms is increasing in radiobiology research. In this study, a previously published mouse phantom described in a previous publication was selected to simulate the process by which radiation passes through the skin, bones, and other organs of an animal to reach the tumor site. The phantom includes adjustable skeletal, lung, and organ cavities with linear X-ray radiation attenuation coefficients that closely approximate those of mouse tissues. In addition, the mouse phantom is open source and costs approximately EUR 100 to fabricate, facilitating its widespread adoption by different research groups [14]. To be able to place the 3D models in the mouse phantom while maintaining sterility, a resin box was designed and 3D printed using materials identical to those used to create the mouse phantom. This allowed for the quick and efficient transfer of the 3D models from the cell culture plate to the phantom for radiation therapy.

While 2D cell culture is the established norm, 3D-printed organ models offer the potential of superior models, though their advantages need to be carefully elucidated. A spatially and temporally controlled extracellular environment, which is essential to accurately model the in vivo environment, is not provided by 2D in vitro cancer cell cultures. Monolayer cell cultures lack the tissue architecture and organization characteristic of native tissues. This limitation makes 2D cancer cell cultures less reliable for studying complex processes within the tumor microenvironment [25]. As demonstrated in this study, a method was developed to simulate realistic lesions in lung cancer patients by printing tumor cells in the center, surrounded by normal primary lung fibroblasts on the outer layer. Both regions were exposed to the same X-ray radiation, allowing the cytotoxic effects of radiotherapy to be clearly visualized by live-dead staining results. Compared to separate irradiation of each cell type in 2D cultures, the images from the 3D model provide a more realistic physiological representation. Although the experimental results for DNA damage and cellular apoptosis from 2D cell cultures and 3D models cannot be directly compared due to their inherent differences, both show a consistent overall trend. However, there are notable differences in many details. Specifically, both types of 2D cells exhibited a high number of γH2AX foci 24 h after irradiation, whereas this effect was less pronounced in lung fibroblasts within the 3D model. A similar pattern was observed with respect to apoptotic effects. The reasons for these observations are complex and require further investigation. First, radiation must pass through the mouse phantom and the hydrogel surrounding the cells in the 3D model, resulting in a slight attenuation of the radiation. In addition, interactions between cell types within the 3D model may also contribute to the observed differences. Taken together, this approach more closely mimics physiological phenomena and better reflects the clinical efficacy of radiation therapy.

Despite the inherent difficulties in making direct comparisons with animal model results due to differences in experimental methods, some similarities can nevertheless be identified. For example, Yin et al. employed BALB/c nu/nu male nude mice for targeted radiotherapy of NSCLC and H&E staining of mouse tissues provided a clear delineation of necrotic and viable tumor areas [26]. This is analogous to the distinct delineation of tumor and normal cells observed in the live/dead staining of the 3D model illustrated in Figure 3g. Furthermore, both 3D tumor models and animal models can withstand multiple rounds of irradiation for extended periods, which is challenging to achieve with 2D cell cultures. In addition, Wedemann et al. examined alterations in the vascular architecture of NSCLC xenograft mouse models subjected to chemoradiotherapy. The researchers evaluated endothelial cell density by CD31 monoclonal antibody staining of vascular tissue sections [27]. In this regard, it is important to note that current 3D tumor models have not yet reached the level of complexity observed in animal models, particularly with respect to the interaction of multiple organ systems. These aspects represent crucial areas for future development in the field of biological 3D printing.

It is also important to acknowledge that responses to radiotherapy are complex and occur at different time points. For example, DNA double-strand breaks frequently occur shortly after radiation [28,29,30], followed by a period of cellular self-repair and a reduction in γH2AX foci. However, apoptotic processes typically require longer periods of time [30]. The limitations of short culture durations in 2D cell cultures, particularly due to the rapid proliferation of tumor cells, for conventional in vitro assays, with the exception of colony formation assays, complicate long-term studies. One of the main advantages of bioprinted tissue models compared to 2D cell culture is the possibility of cultivating the 3D models over a long period of time. In a previous study, we cultured a bioprinted organ model over a period of one month without loss of viability [22]. It will therefore be possible to study the sub-acute effects of irradiation or to investigate the effects of fractionated irradiation.

The use of 3D bioprinting technology to construct NSCLC models for radiotherapy research is currently uncommon in the field of lung cancer treatment. This study has several limitations and represents an area for future development. For example, experimental techniques specifically designed for the characterization of radiation response, such as the clonogenic assay to assess clonogenic survival and thus radiosensitivity and methods to detect cell senescence and apoptosis, are not yet fully implemented for 3D models. Due to various limitations, including the difficulty of observing cell migration and aggregation within hydrogels, these aspects remain challenging.

However, the use of 3D models instead of 2D cell cultures offers new experimental possibilities with regard to the latest developments in the radiotherapy of NSCLC. The rapid proliferation of tumor cells leading to local hypoxia is a significant distinction between tumor cells and surrounding normal tissues. Both physical–chemical and biological processes contribute to the reduced sensitivity of hypoxic tumor cells to ionizing radiation and resistance to hypoxia-related therapies [31]. Therefore, research focusing on cellular hypoxia has increasingly become a new area of interest in the field of radiotherapy for NSCLC. For example, hypoxia and acidity influence cancer cells by modulating immune checkpoint molecules and releasing type I interferons, which allows cancer cells to evade immune surveillance. Targeting hypoxia and acidity may enhance the efficacy of immune checkpoint inhibitors in NSCLC [32].

The present study focuses on the generation of a lung cancer model. The technology, however, can easily be transferred to other types of tumors. We have recently created a perfused model to study liver metastases and the activity of cytotoxic drugs [33]. In this study, we implemented spheroids from various tumor types (including breast cancer, cervical cancer, and neuroblastoma) into the bioprinted hepatic tissue. We assume that it will also be easy to integrate printed models of other tumor types into the mouse phantom, which will expand the field of application of the technology described here for radiotherapy research.

The physiological relevance of the cancer models can be further improved by the inclusion of immune cells. We have recently printed a multilayer lung model for infection studies containing monocytic THP-1 cells, which were differentiated into macrophage-like cells [22]. It will thus be possible to study immune function following radiotherapy in improved versions of the model including immune cells.

In conclusion, this study demonstrates a promising alternative to traditional animal models in radiotherapy research by using a micro-extrusion bioprinting approach to create three-dimensional human lung cancer models and placing them in a mouse phantom.

## 4. Materials and Methods

### 4.1. Cell Culture

Human NSCLC cells (A549; CCL-185, ATCC, Manassas, VA, USA) and normal human primary lung fibroblasts (NHLFb; PCS-201-013, ATCC) were cultured in DMEM high glucose (Biowest, Nuaillé, France) supplemented with 10% fetal bovine serum (FBS; c.c.pro, Oberdorla, Germany), 1% L-glutamine (Biowest), and 1% penicillin/streptomycin (Biowest). Cells were maintained at 37 °C and 5% CO_2_ in a humidified atmosphere. At confluence, cells were washed with phosphate-buffered saline (PBS; Biowest) and harvested using TrypLE Express (Thermo Fisher Scientific, Waltham, MA, USA). For the 2D cell culture experiments, 10,000 (48-Well) and 5000 (96-Well) NHLFb cells or A549 cells were seeded, respectively.

### 4.2. Three-Dimensional Bioprinting

The bioink stock consisted of 6% (*w*/*v*) sodium alginate (Sigma-Aldrich, Steinheim, Germany) and 6% (*w*/*v*) type A gelatin (Sigma-Aldrich) powder that was dissolved in media with supplements on a magnetic stirrer at 1250 min^−1^, at 37 °C overnight. For printing, the bioink stock was heated to 37 °C and loaded into one syringe, while 1.2 M CaSO_4_ solution, media, and NHLFb or A549 cells were loaded into a second syringe, respectively. The two syringes were connected via a Luer lock adapter and thoroughly mixed using an automated mixer at a mixing speed of 10 mm/s for 80 cycles [34]. The final cell-loaded bioinks were composed of 3% (*w*/*v*) alginate, 3% (*w*/*v*) gelatin, and 4% (*w*/*v*) 1.2 M CaSO_4_ and contained 6600 NHLFb/mL or 20,000 A549/mL, respectively. After mixing, the bioinks were crosslinked in a constant temperature chamber at 23 °C for 15 min before being transferred to the printing cartridge. The 3D model was designed by the computer-aided design (CAD) software Rhino7 (Robert McNeel and Associates, Barcelona, Spain) and extruded through a 22G needle at 20–30 kPa using the BioX pneumatic extrusion printer (Cellink, Gothenburg, Sweden). After printing, the constructs were washed with PBS for 10 min and then transferred to a culture medium containing 20 mM CaCl_2_ for long-term culture.

### 4.3. Model System Setup and Treatment Planning

To maintain sterility while placing the 3D model inside the mouse phantom, a resin box was designed using Rhino 7 software. The box was printed using a Formlabs Form 2 3D printer (Formlabs, Somerville, MA, USA) with clear resin V4 material (Formlabs). After printing, the box was cleaned to remove any residual resin and then soaked in deionized water for one month, with the water being changed daily.

A previously published mouse phantom mimicking real mice was used for the radiation experiments [14]. The phantom was manufactured using a stereolithography 3D printing process, which utilized a clear resin and involved manual post-processing steps, such as filling hollow structures with a bone surrogate material. The phantom consists of two halves with tissue surrogates for soft tissue, various organs, and bone, and includes slots for inserting probes in the brain and chest. Prior to the radiation experiment, the 3D lung cancer model was removed from the culture medium and placed in the resin box, which was then positioned in the chest slot of the mouse phantom (Figure 1d).

The Eclipse External Beam Planning software (version 15.6, Varian Medical Systems, Palo Alto, CA, USA) was used as the treatment planning system (TPS). First, the two CTs of the 3D phantom and the in vivo mouse model [16] were imported into the TPS. Next, the CT of the in vivo mouse model was adjusted so that the position of the tissue box in the 3D phantom correlated with the position in the other CT so that the structure of the tissue box could be transferred. In this way, both the density and the dose distribution in the corresponding CT slices could be compared later.

For dose calculation, a 5 cm rectangular field was selected with the isocenter directly in the center of the tissue box in both CTs. The irradiation energy used was 6 MeV photons with a fractional dose of 2 Gy. The dose in the irradiation plan for the 3D phantom was normalized so that the tissue box received 100% of the said 2 Gy in the arithmetic mean for the whole volume. To maintain comparability, the irradiation plan for the in vivo mouse model was given the exact same parameters. A tool in TPS was used to analyze the density of both CTs. Slices in each CT were selected such that the positions of the tissue boxes correlated with one another. The ROI (red square) was then selected for density analysis and the tool provided a histogram (Figure 2). For dose distribution analysis, the same CT slices were viewed side by side. This allowed a comparison of the isodose lines.

### 4.4. Irradiation

The 2D cells were cultured for 24 h after seeding, and the 3D models were cultured for one week after printing before being subjected to radiation tests with and without the phantom with doses of 4 and 8 Gy irradiation using the YXLON MaxiShot X-ray device (YXLON International X-Ray GmbH, Hamburg, Germany) with the following settings: 200 kV, 15 mA, 5.5 FOC, and dose rate 0.89 Gy/min. Medium exchange was performed after irradiation on day 2. Sham-irradiated (“0 Gy”) models were used as negative controls and were also transported to the irradiation device to control for possible temperature and atmosphere changes.

### 4.5. Cell Viability Assay

Cell viability assays were performed using the LIVE/DEAD viability/cytotoxicity kit (Thermo Fisher Scientific) according to the manufacturer’s instructions. Briefly, the 2D cells or 3D models were analyzed by fluorescence microscopy (Observer Z1, Zeiss, Jena, Germany) 1 h after incubation in phenol red-free RPMI medium (Biowest) containing 2 µM calcein-AM and 4 μM ethidium homodimer-1.

### 4.6. Metabolic Activity Assay

Cell viability was determined by tetrazolium hydroxide salt (XTT) assays (Alfa Aesar, Ward Hill, MA, USA) according to the manufacturer’s instructions. For this, 200 µL of 1 mg/mL XTT reagent and 100 µM phenazine methosulphate (PMS; AppliChem, Darmstadt, Germany) in phenol red-free RPMI medium were added to the 2D cells in 48-well plates and incubated for 4 h at 37 °C and 5% CO_2_. Then, 100 µL of supernatant from each well was transferred to a 96-well plate and absorbance was measured at 450 nm and 620 nm reference using a Sunrise microplate reader (Tecan, Männedorf, Switzerland). For 3D constructs, the XTT/PMS solution was added to the bioink in a 5-fold excess.

### 4.7. Immunofluorescence Staining of 2D Cells and 3D Models

For immunofluorescence staining, the 3D models were fixed in 3.7% formalin (Carl Roth, Karlsruhe, Germany) for 15 min and washed three times with HBSS (Biowest). After dehydration, the constructs were embedded in paraffin and sectioned at 16 µm using a microtome (pfm Rotary 3004 M, pfm medical, Cologne, Germany). Following deparaffinization, rehydration, antigen retrieval, and blocking, the sections were incubated overnight at 4 °C with the appropriate primary antibodies against gamma H2A.X (1:250; ab81299, Abcam, Cambridge, UK), cleaved caspase-3 (1:400; #9661, Cell Signaling Technology, Danvers, MA, USA), and pan-cytokeratin (1:400; ab27988, Abcam). The samples were then washed three times with HBSS and incubated with appropriate Alexa 546 and Alexa 488 conjugated secondary antibodies (Thermo Fisher Scientific) for 1 h at room temperature. After incubation, samples were washed twice with HBSS, stained with 1 µg/mL 4′,6-diamidino-2-phenylindole (DAPI, Sigma-Aldrich) for 30 min to visualize nuclei, and then washed again with HBSS. Stained samples were analyzed by fluorescence microscopy (Observer Z1).

Two-dimensional cells were first washed with HBSS and then fixed with 3.7% formalin for 10 min at room temperature. After fixation, samples were washed twice with HBSS, blocked, and treated as described above.

### 4.8. Cytotoxicity Assay

Cytotoxicity assays were performed using the LDH Cytotoxicity Assay (Thermo Fisher Scientific) according to the manufacturer’s instructions. Briefly, 2D cells were cultured in 100 µL of medium in a 96-well plate while 3D models were cultured in 1 mL of medium in a 24-well plate. The absorbance of the collected and processed 50 µL supernatants was measured at 490 nm and 680 nm using a Sunrise microplate reader.

### 4.9. Statistics

Results are presented as the mean ± standard deviation (SD) of at least three independent experiments. Statistical analysis was performed using GraphPad Prism 7 software (Dotmatics, Boston, MA, USA). For comparisons between two groups, an unpaired two-sample *t*-test was used for analysis. One-way ANOVA was used for the analysis of variance to compare multiple groups. Statistical significance was accepted at the levels of * *p*  ≤  0.05, ** *p*  ≤  0.01, *** *p*  ≤  0.001, and **** *p*  ≤  0.0001.

## 5. Conclusions

In conclusion, the study demonstrates that using a micro-extrusion bioprinting approach to create a three-dimensional human lung cancer model offers a promising alternative to traditional animal models in radiotherapy research. By embedding lung tumor cells in human primary lung fibroblasts and placing them in a mouse phantom, the model effectively mimics the physiological conditions and radiation attenuation of real tissues. The selective cytotoxic effects of X-rays on tumor cells, along with differential responses in metabolic activity, cell death, apoptosis, and DNA damage, highlight the model’s potential to accurately reflect therapeutic outcomes. This innovative setup not only addresses ethical concerns associated with animal testing but also provides a more relevant and accurate tool for studying radiation effects and developing new treatment options for lung cancer.

## Data Availability

The raw data supporting the conclusions of this article will be made available by the authors upon request.

## Supplementary Materials

The following supporting information can be downloaded at https://www.mdpi.com/article/10.3390/ijms251910268/s1.
