# Supplementary material for: Three-Dimensional-Bioprinted Non-Small Cell Lung Cancer Models in a Mouse Phantom for Radiotherapy Research
Source: Int J Mol Sci. 2024 Sep 24;25(19):10268. doi: 10.3390/ijms251910268 (PMC11476964; doi:10.3390/ijms251910268)
Supplement: Supplementary file 1 [file ijms-25-10268-s001.zip › ijms-3197167-supplementary.pdf]

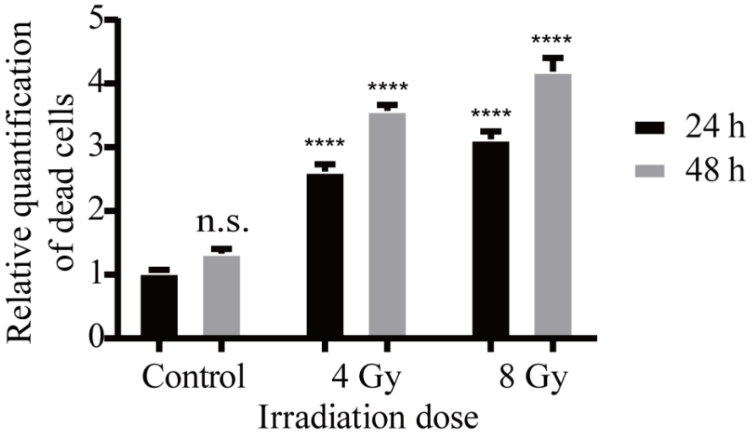

Figure S1. Quantification of dead cells in the 2D cell viability stainings 24 and 48 hours after irradiation (Figure 3). Quantitative analysis of red fluorescent cells was performed using ImageJ 1.53e to compare the number of dead cells in the field of view. Values were normalized to the number of dead cells in the control group 24 hours after irradiation. Data from three independent experiments are presented as mean ± standard deviation, **** $p < 0.0001$.

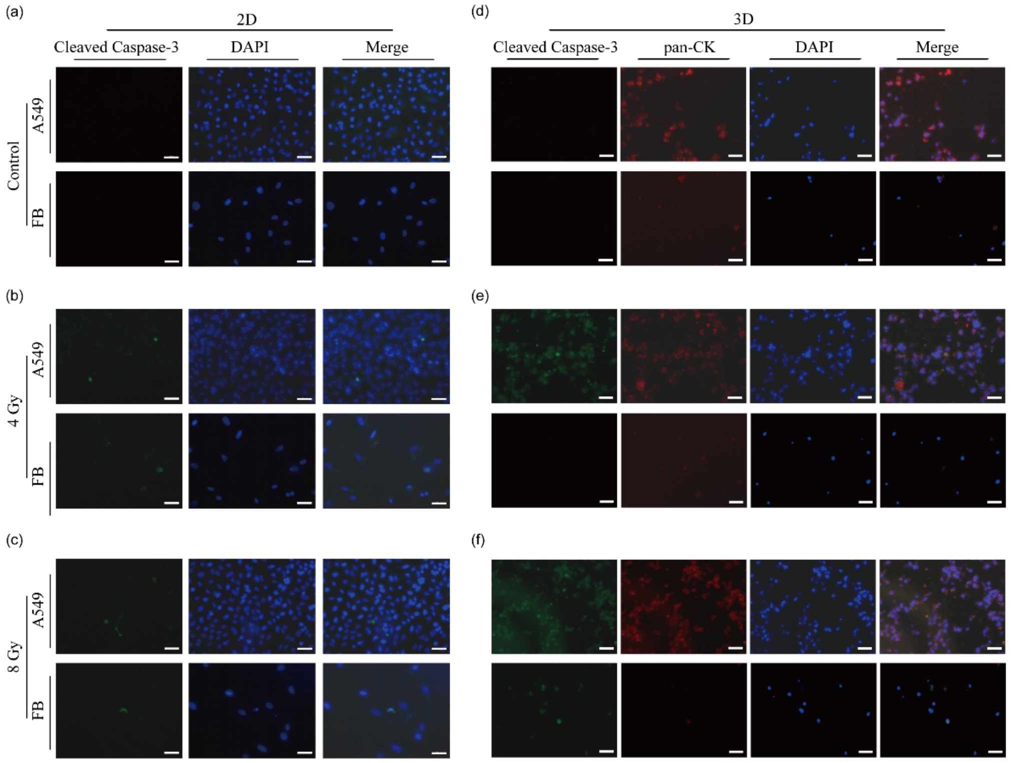

Figure S2. Cleaved caspase-3 immunofluorescence staining of irradiated 2D cells and 3D models. (a-c) Immunostaining images of A549 cells and FB from non-irradiated control (a), 4 Gy irradiation group (b) and 8 Gy irradiation group (c). 2D cells were fixed 24 hours after irradiation. Cleaved caspase-3 staining (green channel) indicates apoptosis induction. DAPI was used for nuclear counterstaining (blue channel). Scale bar: 20 µm. (d-f) Immunohistochemical staining images of 3D

lung cancer models from the non-irradiated control group (d), 4 Gy irradiation group (e) and 8 Gy irradiation group (f). The models were fixed, dehydrated, and paraffin-embedded 24 h after irradiation, followed by sectioning at 16 mm thickness. The sections were then subjected to immunostaining. Cleaved caspase-3 staining (green channel) indicates apoptosis induction. The samples were also stained with antibodies against pan-cytokeratin (pan-CK) to confirm their identity as epithelial cells (red channel). DAPI was used for nuclear counterstaining (blue channel). Scale bar: 20 μm.

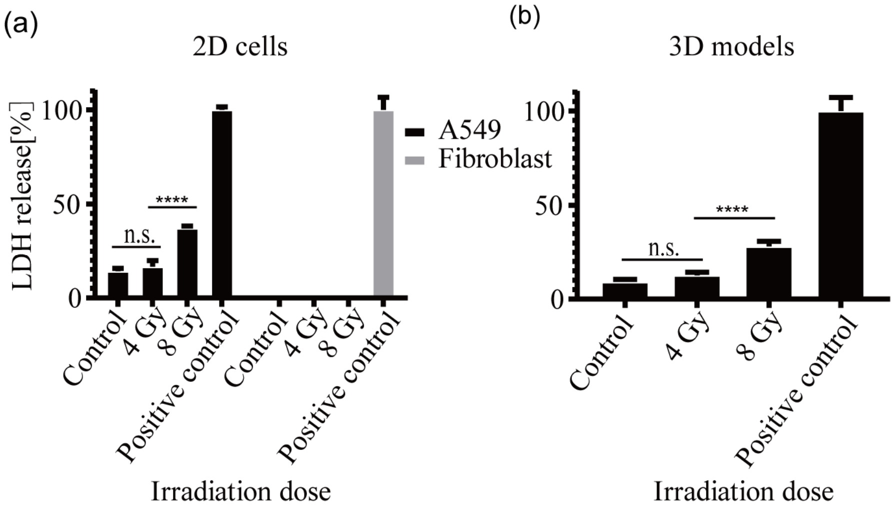

Figure S3. LDH release of 2D cells and 3D models 24 hours after radiotherapy. (a) Relative LDH release in 2D cells 24 hours after irradiation, with LDH release from lysis buffer-treated cells used as a positive control. All data are presented as the mean ± standard deviation of at least three independent experiments; ****$p < 0.0001$. (b) Relative LDH release in 3D models 24 hours after radiotherapy, with LDH release from lysis buffer-treated cells used as a positive control. All data are presented as the mean ± standard deviation of at least three independent experiments; ****$p < 0.0001$.
